# Transport of non-classical light mediated by topological domain walls in a SSH photonic lattice

**DOI:** 10.1038/s41598-024-63321-3

**Published:** 2024-05-30

**Authors:** Gabriel O’Ryan Pérez, Joaquín Medina Dueñas, Diego Guzmán-Silva, Luis E. F. Foa Torres, Carla Hermann-Avigliano

**Affiliations:** 1 Departamento de Física, Facultad de Ciencias Físicas y Matemáticas, Universidad de Chile, Santiago, Chile; 2 Millenium Institute for Research in Optics (MIRO), Santiago, Chile; 3 ICN2-Institut Català de Nanociència i Nanotecnologia, Campus UAB, 08193 Bellaterra, Barcelona Spain; 4 Department of Phyics, Universitat Autónoma de Barcelona (UAB), Campus UAB, 08193 Bellaterra, Barcelona Spain

**Keywords:** Quantum optics, Photonic devices, Electronic properties and materials

## Abstract

Advancements in photonics technologies have significantly enhanced their capability to facilitate experiments involving quantum light, even at room temperature. Nevertheless, fully integrating photonic chips that include quantum light sources, effective manipulation and transport of light minimizing losses, and appropriate detection systems remains an ongoing challenge. Topological photonic systems have emerged as promising platforms to protect quantum light properties during propagation, beyond merely preserving light intensity. In this work, we delve into the dynamics of non-classical light traversing a Su-Schrieffer-Heeger photonic lattice with topological domain walls. Our focus centers on how topology influences the quantum properties of light as it moves across the array. By precisely adjusting the spacing between waveguides, we achieve dynamic repositioning and interaction of domain walls, facilitating effective beam-splitting operations. Our findings demonstrate high-fidelity transport of non-classical light across the lattice, replicating known results that are now safeguarded by the topology of the system. This protection is especially beneficial for quantum communication protocols with continuous variable states. Our study enhances the understanding of light dynamics in topological photonic systems and paves the way for high-fidelity, topology-protected quantum communication.

## Introduction

Photonics plays a fundamental role in a wide array of technological applications, including long-distance telecommunications^1^ and precision laser-based sensors^2^. It serves as the backbone for systems that rely on light as the medium for information transmission. Recent years have witnessed significant advancements in the field of quantum photonics^3^. These systems harness photons’ capacity to retain quantum properties, even at room temperature^4^, resulting in minimal noise levels and limited susceptibility to environmental interference. These advantages hold great promise for applications in communication, computation, and simulation, particularly using quantum light^5,6^. Nevertheless, challenges like error accumulation, decoherence, and the lack of precise control of quantum states present substantial hurdles, particularly when striving for secure, stable, and long-distance quantum computing.

Waveguides arrays emerge as a dependable, high-speed, and cost-effective platform to transport radiation^7,8^. Light is confined to travel within individual waveguides, with a probability of transitioning to their neighbor guides depending on different parameters such as geometry and index of refraction. The challenges encompass dispersion and losses during propagation and inefficient light coupling to the waveguides. These are highlighted when the objective is to preserve quantum properties of light^9^.

Topological systems may be considered as a natural scenario to propagate quantum light more robustly, since it offers robustness in comparison to topologically trivial arrays^10^. Topology in a photonic array influences the quantum characteristics of light, extending beyond the qualities of localization and robustness often associated with classical light (intensity). Quadrature protection of squeezed light and enhanced teleportation protocols have been proposed^11^, which is remarkably important when performing quantum information protocols with continuous variables^12^.

In this work, we study a Su-Schrieffer-Heeger (SSH) photonic lattice with domain walls (DW). This array has been deeply studied due to its simplicity yet clear topological nature, making it a suitable candidate to study topological effects on new platforms^13–17^. The concept of a domain wall is widely employed to denote the boundary between two different behaviors^18^. In our context, when two systems in a different topological phase are connected, a domain wall is created on the junction and the number of states on it is determined by the difference between topological invariants^19^. We re-positioned the DWs across the lattice through a local modulation of the coupling between waveguides. Light injected into the DW follows its movement. Moreover, we can approach the DWs as much as needed, until they start to interact, forming effective couplers such as a dimer. The system is fine-tuned to minimize dispersion during modulation, which prevents the loss of the initial state. Our findings show how topological properties bestow robustness to quantum light while it propagates between DWs.

## SSH lattice with domain walls

**Figure 1:**
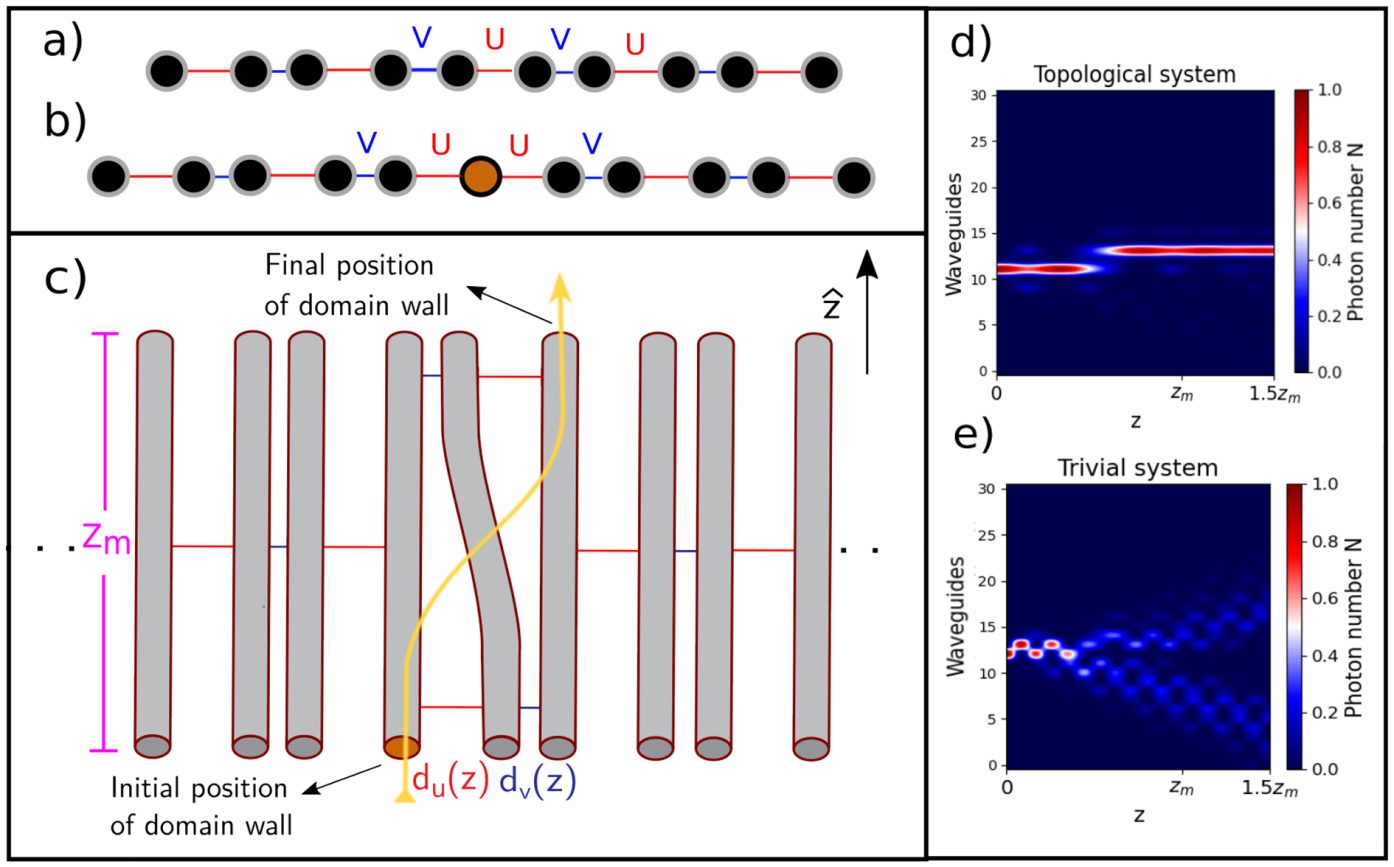
(**a**) SSH array with intra-coupling *u* and inter-coupling *v*. (**b**) SSH array with a topological domain wall. (**c**) Example of how the bending is done. The distance between waveguides depends on the propagation and the whole process has a distance of $Z_{m}$ called the modulation length. This is the principal mechanism to transport light across our lattice. (**d**) Shows the photon number evolution through the bending in a topological domain wall. (**e**) Shows the photon number evolution through the bending in a trivial domain wall.

The SSH model^19,20^ consists of a one-dimensional dimerized chain, as depicted in Fig. 1a. The unit cell possesses two sites with intra-coupling *u* and inter-coupling *v*. Defining $\delta =|v/u|$, if $\delta <1$, the system is in a trivial phase but if $\delta >1$, the system is in a topological phase, and two zero-energy states appear within the energy gap (See Supplemental Information for more details). These states are exponentially localized on the edges, possess sub-lattice symmetry, and are robust to coupling disorder^19^. By either repeating coupling *u* or *v* inside the array, a domain wall is added—which is equivalent to joining two topological systems with different edges—as depicted in Fig. 1b. The Hamiltonian of the system is

1 $$\begin{aligned} \hat{\mathcal {H}} = \sum _{n\le m} (u \hat{a}_{n} \hat{b}^{\dagger }_{n} + v \hat{b}^{\dagger }_{n-1}\hat{a}_{n}) + \sum _{n>m}(v\hat{a}_{n}^{\dagger }\hat{b}_{n} + u \hat{b}^{\dagger }_{n-1} \hat{a}_{n}) + h.c., \end{aligned}$$

where $a^\dagger _n(a_n)$ and $b^\dagger _n(b_n)$ are the creation (annihilation) operator at site *n* of the corresponding unit cell and it is assumed there is only one domain wall in site *m* with coupling *u*. Although either coupling *u* or coupling *v* can be used in the interface to create a domain wall, we will study the one with coupling *u*, because it allows having an isolated state on the domain wall. The DW state shares the topological properties of the edge state and it hybridizes with the nearest edge if it is in a topological phase. This provokes different dynamics compared to a domain wall in a trivial phase as seen in Fig. 1d,e. The propagation of light inside both types of domain wall was studied in^21^. A third possible case is when the DW is formed between a trivial and a topological system. Interestingly, in this case the DW shows the same dynamics as the case of a DW between two topological systems. This scenario is studied in Supplemental Information.

### Guiding non-classical light through topological domain walls

Given the Hamiltonian in Eq. (1), it is possible to find the infinitesimal unitary operator $\hat{U}(z+dz)$ which can be used to obtain the entire evolution, even for time-dependent Hamiltonians (See Supplemental Information for more details). The operator $\hat{U}$ depends on the propagation distance *z* (analog to time in condensed matter systems). For this task, we use a trotter algorithm^22^ which calculates the light amplitude of every waveguide in an iterative way for each dz. Here we explore the properties of the array when propagating quantum states of light. We consider four initial states $\left| \psi _{0}\right\rangle$ in the DW: single photon, coherent, vacuum single-mode squeezing, and vacuum two-mode squeezing states. As pure states, they can be described as operators acting on the vacuum^23^.

2 $$\begin{aligned}&\left| \psi _{0}\right\rangle = \hat{a}^{\dagger }_{DW} \left| 0\right\rangle = \left| 1\right\rangle \end{aligned}$$

3 $$\begin{aligned}&\left| \psi _{0}\right\rangle = \hat{D}_{DW}(\alpha )\left| 0\right\rangle = \left| \alpha \right\rangle \end{aligned}$$

4 $$\begin{aligned}&\left| \psi _{0}\right\rangle = \hat{S}_{DW}(\xi ) \left| 0\right\rangle = \left| \xi \right\rangle \end{aligned}$$

5 $$\begin{aligned}&\left| \psi _{0}\right\rangle = \hat{S}_{0,DW}(\xi ) \left| 0\right\rangle = \left| \xi _{0,DW}\right\rangle \;, \end{aligned}$$

where $\hat{a}^{\dagger }_{DW}(\hat{a}_{DW})$ is the creation (annihilation) operator on the position of the DW, $\hat{D}_{DW}(\alpha ) = \exp {(\alpha \hat{a}_{DW}^{\dagger } - \alpha ^{*}\hat{a}_{DW})}$ the displacement operator on the DW , $\hat{S}_{DW}(\xi ) = \frac{1}{2}[\xi ^{*} \hat{a}_{DW}^{2} - \xi (\hat{a}_{DW}^{\dagger })^{2}]$ the single-mode squeezed operator on the DW and $\hat{S}_{0,DW}(\xi ) = \frac{1}{2}(\xi ^{*} \hat{a}_{DW}\hat{a}_{0} - \xi \hat{a}_{DW}^{\dagger }\hat{a}_{0}^{\dagger })$ the two-mode squeezed operator between the edge and the DW. We choose the edge as the other half because the edge state has the same energy as the DW state. The amplitude of the states is computed by taking the mean value of the photon number operator

6 $$\begin{aligned} \langle \hat{N}_{i,j}(z)\rangle = \left\langle \psi (z)\right| \hat{a}_{i}^{\dagger }\hat{a}_{j}\left| \psi (z)\right\rangle . \end{aligned}$$

To study squeezed light, we use the usual decibel function that compares the state’s uncertainty with the one of a coherent state,

7 $$\begin{aligned} S_{i}^{(1,2)}(z,\phi )[\text {dB}] = 10 \log _{10} \Big ( \frac{\left\langle \psi (z)\right| \Delta \hat{X}^{(1,2)}_{i}(\phi )\left| \psi (z)\right\rangle ^{2}}{\langle \Delta \hat{X}^{(1,2)}_{i} \rangle ^{2}_{coh}} \Big ), \end{aligned}$$

with $\langle \Delta \hat{X}\rangle ^{2} = \langle \hat{X}^{2}\rangle - \langle \hat{X} \rangle ^{2}$ the standard deviation. For this we consider the generalized quadrature as

8 $$\begin{aligned} \hat{X}_{i}(\phi ) = \frac{1}{2}(\hat{a}_{i}e^{i\phi } + \hat{a}^{\dagger }_{i} e^{-i\phi } ). \end{aligned}$$

The two orthogonal quadratures are then $\hat{X}^{(1)}_{i}(\phi ) = \hat{X}_{i}(\phi )$ and $\hat{X}^{(2)}_{i}(\phi ) =\hat{X}_{i}(\phi + \frac{\pi }{2})$ and the two-mode quadrature is $\hat{X}^{(1,2)}_{ij} = \frac{1}{\sqrt{2}}\big ( X^{(1,2)}_{i}(\phi ) + X^{(1,2)}_{j}(\phi )\big )$, where $\phi$ is the phase of the quadrature. We can study the dynamics of the squeezing by computing the uncertainty of both orthogonal quadratures. For simulations and comparisons between the states, we set the mean photon number equal to 1, meaning that for the coherent state $\alpha =1$ and for the squeezed state $\xi ={{\,\textrm{arcsinh}\,}}(1)$ (7.65 [dB] in squeezing magnitude). We propose a method to move domain walls across the lattice by slowly shifting two adjacent couplings. In photonic lattices, this can be achieved by bending one waveguide, as shown in Fig. 1c. This moves the domain wall to the next adjacent waveguide, either to the left or right, depending on the direction of curvature. The objective is to guide the light and transport it to its new position with minimum dispersion, which depends on how we perform the bending (how fast, how sharp). We observe that the light propagates across domain walls only when the array is in the topological phase (see Fig. 1d), this is because the hybridization to the edge lowers the impact of the transfer on the state’s energy, keeping it inside the energy gap and away from the dispersive bands. If we make a DW using the coupling *v* and then switch the couplings so that $\delta < 1$, we get a DW between coupling *u* but in a trivial phase. In this trivial case, the initial input just spreads along the array if we try to transfer it as shown in Fig. 1e due to the interaction of the state with the dispersive bands. To present our system as an experimentally feasible one, we assume realistic values and limits. The coupling *C* is related to the distance *D* between waveguides as $C = c_{2} \exp (- c_{1}D)$, with the constants $c_{1,2}$ depending on the parameter of fabrication^24^. The main parameters to consider are the coupling ratios $\delta$, the modulation length $Z_{m}$, and the slope *s* of the bending. Logistic function or s-shape functions are usually picked for activation or adiabatic processes due to their expected suppression of leakage^25,26^. Among this family, we pick the function

9 $$\begin{aligned} f(z) =A- \frac{B \exp (-Z_{m}/z)}{ s \exp [-1/(1-z/Z_{m})] + \exp (-Z_{m}/z)}\;. \end{aligned}$$

A slight variation is used in Ref.^27^ resulting in low leakage. The constants *A* and *B* are obtained by imposing the initial coupling difference while *s* controls the slope. The *s* parameter is optimized to give the greatest transmission given the coupling difference and modulation length. It also serves a direct variable to change the radius of curvature. While many parameters fit the boundary conditions, not all of them are realistic. Previous works show that bending waveguides introduce radiation losses proportional to the radius of curvature, meaning that a sharper bend generates more losses^28,29^. This imposes a limitation if the curved waveguide is guiding light. Remarkably, our model does not have this limitation, because curved waveguides only serve as a bridge between straight waveguides, allowing to spatially move each DW. For the following sections, we consider a lattice of 32 waveguides and parameters (see Fig. 1) $d_{u} = 22~\upmu$m, $d_{v} = 10 ~\upmu$m , $Z_{m} = 5.5$ cm and $s = 1.5$. The couplings used are $u = 0.69~\text {cm}^{-1}$ , $v = 3.22~\text {cm}^{-1}$ and $\delta = 4.62$ (see Supplemental Information for more details about this lattice and how this set of parameters was chosen).

**Figure 2:**
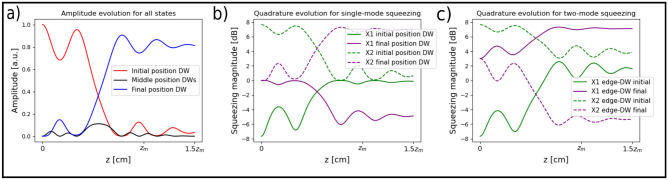
(**a**) Shows the photon number evolution for the four initial states (Eqs. 2–5) across the waveguides, from the initial to the final position of the DW as depicted in Fig. 1c. (**b**) Shows the single-mode quadrature magnitude evolution of the single-mode squeezed state when moving the DW. (**c**) Shows the two-mode squeezed quadrature magnitude evolution between the edge and the moving DW.

The photon number evolution for all initial states is shown in Fig. 2a. All states show the same *N*(*z*) evolution, which is expected in waveguides systems because *N* evolves in the same way as intensity distribution of classical light^30,31^ and we have treated it as a closed system. Quantum properties are revealed when correlations between photons are considered, for example, the second-order correlation function $g^{(2)}$^31^. For all the states we considered, $\sim 80\%$ of the initial photon number N could be transported. The 20% remaining is scattered during dynamics due to dispersion to other parts of the system. If the bending is done optimally, the light disperses to the same sublattice as the DW and nothing enters the bent waveguide. After the transport, the light remains localized on the new position. Notice that the DW state maintains its topological properties after movement. This is due to the modulation affects only local couplings which has an impact on the states inside the gap but almost none on the majority of the bulk states, implying that throughout the evolution the gap never closes and hence the topological phase remains the same. Current studies of transport within waveguide lattices have shown that the initially squeezed quadrature rotates^32^ proportional to the transported distance, not including disorder effects. In contrast, in our system, the phase of the initially single-mode squeezed quadrature is maintained during the dynamics as shown in Fig. 2b. The two-mode squeezing presents a similar protection, but in this case, the squeezed quadrature rotates by $\pi /2$ and the correlation with the edge is transported to the new DW (see Fig. 2c). This is due to a phase difference generated by moving the DW but not the edge state. Moving two DWs with a two-mode squeezing between them does not generate this rotation. Then, with this procedure, we can transport non-classical light across two waveguides with transmission higher than 80%. This can be repeated to move the DW to a new position of the same sub-lattice. Just like the amplitude, the initially squeezed quadrature remains localized and locked after the transport ends.

## Topological beam splitter

**Figure 3:**
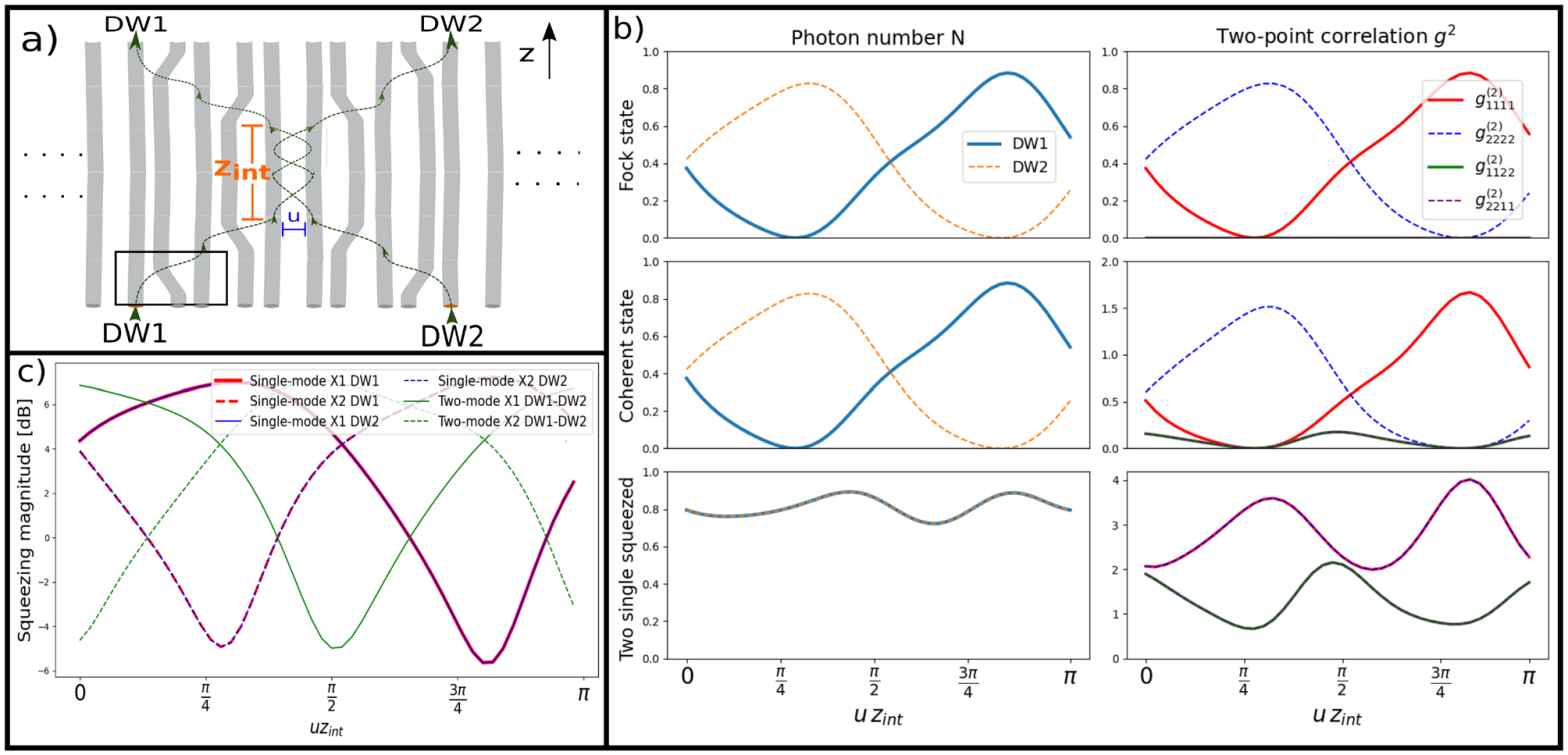
(**a**) Example of the effective beam-splitter. The black box encapsulates the size of one movement. Light is injected on DW1 and DW2, interferes at the middle for a length $z_{int}$ with coupling *u*, and then returns to the same waveguide that was injected on. (**b**) Photon number *N* (left column) and two-point correlation function $g^{2}$ (right column) versus normalized distance $uz_{int}$ for three different input states: Single photon Fock state in DW1, coherent state of amplitude $\alpha =1$ in DW1 and two single-mode squeezed states of amplitude $\xi ={{\,\textrm{arcsinh}\,}}(1)$ in both DWs. (**c**) Shows the evolution of single and two-mode quadratures within and between DWs, respectively.

One interesting point to observe in topological arrays is the interaction between two (or more) topological states since this interaction can perform a primary element of any photonic circuit as it is a beam splitter operation^33^. In our scheme, it is possible to obtain an effective beam splitter with two DW (or more for a multiport splitter), as depicted in Fig. 3a. Light is injected into the moving DWs splitting the signal. After a fixed length, both domain walls are separated one more time into their original waveguides (not mandatory, other waveguides can be used), and the output is then studied. To demonstrate the splitting and transport properties we propagate a coherent state in one DW and vacum in the other $\left| \alpha \right\rangle \otimes \left| 0\right\rangle$, a Fock state $\left| 1\right\rangle \otimes \left| 0\right\rangle$, and two single mode squeezed states $\left| \xi \right\rangle \otimes \left| \xi \right\rangle$ as different input conditions. We analyze the output for different lengths of interaction $z_{int}$, taking in consideration the photon number *N* and the correlation function $g^{(2)}$, both depending on the normalized interaction length

10 $$\begin{aligned}&\hat{N}_{i,j}(u z_{int}) = \langle \hat{N}_{i,j}\rangle = \langle \hat{a}_{i}^{\dagger }\hat{a}_{j}\rangle (u z_{int}) \end{aligned}$$

11 $$\begin{aligned}&g^{(2)}_{i,j,k,l}(u z_{int}) = \langle \hat{N}_{i,j}\hat{N}_{k,l}\rangle (u z_{int}) = \langle \hat{a}_{i}^{\dagger }\hat{a}_{j}\hat{a}_{k}^{\dagger }\hat{a}_{l}\rangle (u z_{int}). \end{aligned}$$

We observed that the output display in Fig. 3b, is analogous to the propagation inside two waveguides (dimer) for the three cases studied here^34,35^. The single photon state oscillates between DWs for both *N* and $g^{2}$ functions, not generating a correlation between waveguides. The coherent state with $\alpha =1$ behaves similarly but has a higher value in the correlation function (diagonal) and presents low off-diagonal correlation values between the DWs at $u z_{int} = (0,\frac{ \pi }{2})$. This is because for the coherent state $\left| \alpha \right\rangle = e^{-|\alpha |^{2}/2} \sum _{0}^{\infty } \alpha ^{j}(j!)^{-1/2}\left| j\right\rangle$, the initial vacuum component does not transfer between waveguides. Hence, for a coherent state with $\alpha \le |1|$, the vacuum amplitude is comparable to the other amplitudes, thus the appearance of off-diagonal correlations^34^. This correlation becomes negligible as the photon number increases. The squeezed states present a constant photon number due to being injected on both DWs. The decrease in the diagonal correlation while increasing the off-diagonal, shows the formation of two-mode squeezing around $u z_{int} = (0,\frac{\pi }{2})$ as shown in Fig. 3c. The states go back to being single mode squeezed light at around $u z_{int} = (\frac{\pi }{4}, \frac{3\pi }{4})$. This behavior oscillates as a function of $uz_{int}$, just as how a dimer should behave with such a non-classical light^35^.

By using DWs we can transport non-classical light across the system and by the interaction of DWs, light can be split in different waveguides. The transmission stays above 80% which is a bit larger than one DW movement transmission. This is because in the case of only one DW, part of the light is dispersed into the array (there is no perfect transmission). In contrast, having two DWs allows the leakage of one DW to be “caught” by the other and vice versa. We also emphasize that the energy band never closes and the system is always in the same topological phase along the whole dynamics. Besides from photon number, the topology protects the initially squeezed quadrature, and transporting the state does not rotate it. This prevention of rotation is crucial as both single-mode states entering the effective dimer must have the same phase to maximize the generation of two-mode squeezed light. Rotation of the quadrature and formation of two-mode squeezing starts just when the effective dimer forms.

### Robustness against disorder

**Figure 4:**
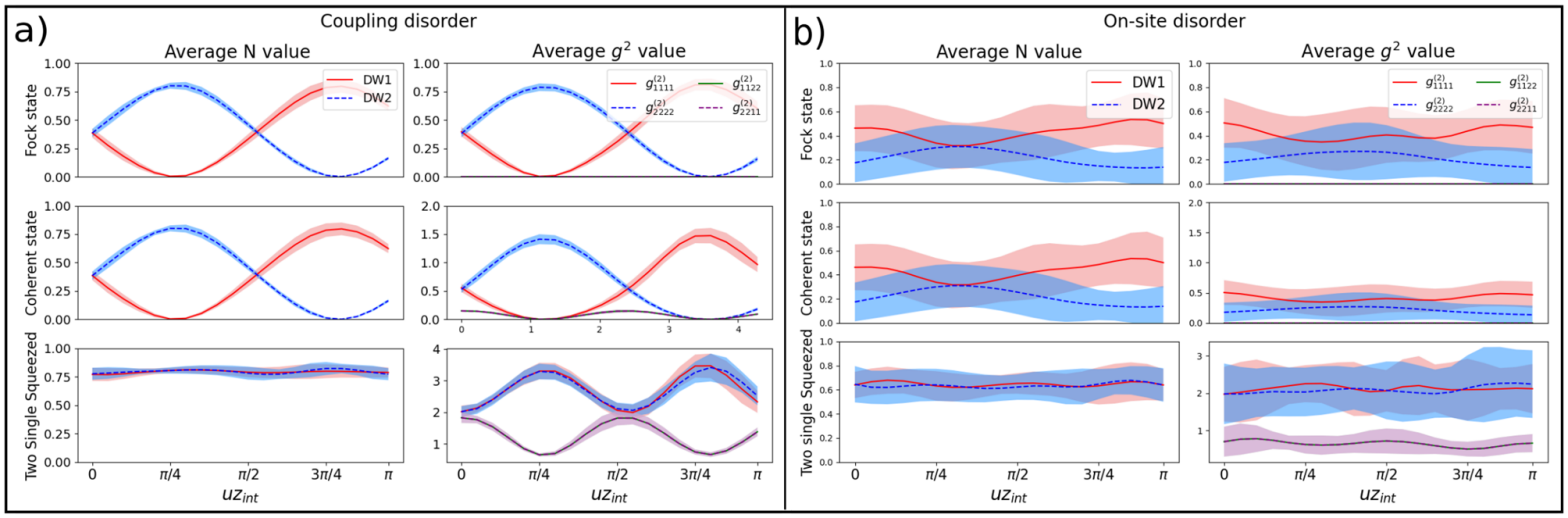
(**a**) Show the average (continuous and dashed lines) and standard deviation (shaded area) for 20 repetitions of the output photon number and two-point correlation function on each DW output as depicted in Fig. 3b when it is subject to a uniform coupling disorder $\Delta =\pm 1.3$ cm^-1^. (**b**) Same as in part (**a**) but for the case of on-site disorder.

We test our system against two kinds of disorders: the off-diagonal disorder, which affects the coupling between waveguides, and the diagonal disorder, which mainly depends on the size and change of refractive index of the waveguide (reflected in the on-site energy parameter). The first one is known to preserve the sub-lattice symmetry, while the second does not. We add a uniform disorder $\Delta$ for both cases, with a value inside the interval of $[-1.3,1.3]$ cm^-1^. We observe that the system is robust against coupling disorder maintaining the output for both N and $g^{(2)}$, as shown in Fig. 4a. The squeezing state is particularly interesting, as discussed before, due to the generation of maximal two-mode squeezing (i.e. without single mode contributions) inside a dimer. Under disorder, a trivial array is likely to fail as the phase varies randomly. Nevertheless, in our system due to the topological protection the state generates almost no rotation of the quadrature preventing decoherence in the squeezed phase and allowing the formation of two-mode squeezed light.

For the case of on-site disorder, the results show a great dispersion (shaded area in Fig. 4) for both *N* and $g^{2}$. This contrasts with the case of off-diagonal disorder, implying a break of sub-lattice symmetry. The $g^2$ correlation function for the squeezed states behaves noticeably worse than the Fock and coherent state even though the decrease in photon number is similar. This is because on-site disorder, apart from destroying the state, rotates the quadrature randomly before arriving at the effective dimer, causing both single-mode states to have a decoherence in the squeezed phase and unable to generate maximal two-mode states.

## Conclusions

We presented a photonic waveguide model featuring topological domain walls capable of guiding light by recursively bending waveguides. Topology emerges as a crucial ingredient for this mechanism to work since a trivial domain wall cannot achieve the same behavior. The quality of the transport can be optimized with lattice parameters such as the propagation length and slope of the bending. We optimize our system, achieving above 80% transmission using realistic experimental values. To test our system, we used Fock states, coherent states, and squeezed states, finding in the latter that topology prevents the initially squeezed quadrature from rotating during transport. The interaction of two domain walls forms an effective dimer acting as a beam-splitter to the injected light. The phase protection during the transport allows the effective dimer to generate two-mode squeezing even in the presence of coupling disorder, contrasting, on-site disorder degrades the photon number and generates decoherence in the squeezed phase. Our protocol can be extended to multi-port splitting by adding more DWs. We think that the ability to transport quantum light, particularly squeezed states, protecting the squeezed quadrature and not just its amplitude will be beneficial in different quantum communications protocols^11^.

### Supplementary Information

Supplementary Information.

## Data Availability

The datasets generated during and/or analyzed during the current study are available from the corresponding author on reasonable request.

## References

[1] Addanki S, Amiri IS, Yupapin P (2018). Review of optical fibers-introduction and applications in fiber lasers. Results Phys..

[2] Shahbaz M, Butt MA, Piramidowicz R (2023). A concise review of the progress in photonic sensing devices. Photonics..

[3] Moody G (2022). 2022 Roadmap on integrated quantum photonics. J. Phys. Photon..

[4] Flamini F, Spagnolo N, Sciarrino F (2018). Photonic quantum information processing: A review. Rep. Progress Phys..

[5] Aspuru-Guzik A, Walther P (2012). Photonic quantum simulators. Nat. Phys..

[6] O’Brien JL (2007). Optical quantum computing. Science..

[7] Davis KM, Miura K, Sugimoto N, Hirao K (1996). Writing waveguides in glass with a femtosecond laser. Opt. Lett..

[8] Nasu Y, Kohtoku M, Hibino Y (2005). Low-loss waveguides written with a femtosecond laser for flexible interconnection in a planar light-wave circuit. Opt. Lett..

[9] Slussarenko S, Pryde GJ (2019). Photonic quantum information processing: A concise review. Appl. Phys. Rev..

[10] Hafezi M, Mittal S, Fan J, Migdall A, Taylor JM (2013). Imaging topological edge states in silicon photonics. Nat. Photonics..

[11] Dueñas, J. M., Pérez, G. O., Hermann-Avigliano, C. & Foà-Torres, L. E. F. Quadrature protection of squeezed states in a one-dimensional photonic topological insulator. *Quantum*. **5**, 526, 10.22331/q-2021-08-17-526 (2021). (**Publisher: Verein zur Förderung des Open Access Publizierens in den Quantenwissenschaften**)

[12] Braunstein SL, van Loock P (2005). Quantum information with continuous variables. Rev. Modern Phys..

[13] Zurita, J., Creffield, C. E. & Platero, G. Fast quantum transfer mediated by topological domain walls. Quantum. 7, 1043. 10.22331/q-2023-06-22-1043 (2023)

[14] Munoz F, Pinilla F, Mella J, Molina MI (2018). Topological properties of a bipartite lattice of domain wall states. Sci. Rep..

[15] Yuan J, Xu C, Cai H, Wang D-W (2021). Gap-protected transfer of topological defect states in photonic lattices. APL Photon..

[16] Meier EJ, An FA, Gadway B (2016). Observation of the topological soliton state in the su-schrieffer-heeger model. Nat. Commun..

[17] Li Y (2022). Effective hamiltonian for photonic topological insulator with non-hermitian domain walls. Phys. Rev. Lett..

[18] Kumar D (2022). Domain wall memory: Physics, materials, and devices. Phys. Rep..

[19] Asbóth, J. K., Oroszlány, L. & Pályi, A. *A Short Course on Topological Insulators: Band-structure topology and edge states in one and two dimensions*, vol. 919 (2016). ArXiv:1509.02295 [cond-mat].

[20] Su WP, Schrieffer JR, Heeger AJ (1979). Solitons in polyacetylene. Phys. Rev. Lett..

[21] Blanco-Redondo A (2016). Topological optical waveguiding in silicon and the transition between topological and trivial defect states. Phys. Rev. Lett..

[22] Poulin D, Qarry A, Somma R, Verstraete F (2011). Quantum simulation of time-dependent Hamiltonians and the convenient illusion of Hilbert space. Phys. Rev. Lett..

[23] Scully MO, Zubairy MS (1997). Quantum Optics.

[24] Szameit A, Dreisow F, Pertsch T, Nolte S, Tünnermann A (2007). Control of directional evanescent coupling in fs laser written waveguides. Opt. Express..

[25] Knapp C (2016). The nature and correction of diabatic errors in Anyon braiding. Phys. Rev. X.

[26] Wiebe N, Babcock NS (2012). Improved error-scaling for adiabatic quantum evolutions. N. J. Phys..

[27] Boross P, Asbóth JK, Széchenyi G, Oroszlány L, Pályi A (2019). Poor man’s topological quantum gate based on the Su-Schrieffer-Heeger model. Phys. Rev. B.

[28] Eaton S (2006). Telecom-band directional coupler written with femtosecond fiber laser. IEEE Photon. Technol. Lett..

[29] Calmano T, Paschke A-G, Müller S, Kränkel C, Huber G (2013). Curved Yb:YAG waveguide lasers, fabricated by femtosecond laser inscription. Opt. Express..

[30] Rai A, Agarwal GS, Perk JHH (2008). Transport and quantum walk of nonclassical light in coupled waveguides. Phys. Rev. A..

[31] Bromberg Y, Lahini Y, Morandotti R, Silberberg Y (2009). Quantum and classical correlations in waveguide lattices. Phys. Rev. Lett..

[32] Swain M, Rai A (2021). Non-classical light in a jx photonic lattice. J. Opt..

[33] Tambasco, J.-L. *et al.* Quantum interference of topological states of light. *Sci. Adv*. **4**, eaat3187. 10.1126/sciadv.aat3187 (2018). **(Publisher: American Association for the Advancement of Science)** 10.1126/sciadv.aat3187 PMC6140626 30225365

[34] Rodrí­guez-Lara, B. M. Propagation of nonclassical states of light through one-dimensional photonic lattices. *JOSA B* **31**, 878–881. 10.1364/JOSAB.31.000878 (2014). **(Publisher: Optica Publishing Group)**

[35] Rojas-Rojas S, Barriga E, Muñoz C, Solano P, Hermann-Avigliano C (2019). Manipulation of multimode squeezing in a coupled waveguide array. Phys. Rev. A..

