# Supplementary material for: Transport of non-classical light mediated by topological domain walls in a SSH photonic lattice
Source: Sci Rep. 2024 May 30;14:12435. doi: 10.1038/s41598-024-63321-3 (PMC11139866; doi:10.1038/s41598-024-63321-3)
Supplement: Supplementary file 1 — Supplementary Information. [file 41598_2024_63321_MOESM1_ESM.pdf]

Supplementary Information for "Transport of non-classical light mediated by topological domain walls in a SSH photonic lattice"

Gabriel O'Ryan[1,2], Joaquín Medina Dueñas[3,4], Diego Guzmán-Silva[1,2],
Luis E. F. Foa Torres[1], and Carla Hermann-Avigliano[1,2]
[1]*Departamento de Física, Facultad de Ciencias Físicas y Matemáticas, Universidad de Chile, Santiago, Chile*
[2]*Millenium Institute for Research in Optics (MIRO), Chile*
[3]*ICN2 - Institut Català de Nanociència i Nanotecnologia, Campus UAB, 08193 Bellaterra, Barcelona, Spain and*
[4]*Department of Phyics, Universitat Autónoma de Barcelona (UAB), Campus UAB, Bellaterra, 08193 Barcelona, Spain*
(Dated: April 30, 2024)

S1. BAND STRUCTURE AND DISTRIBUTION OF STATES

The energy spectrum and distribution of states for a SSH model of 31 sites hosting a domain wall at position 15 and in a topological phase is shown in Figure S1. We define the inverse participation ratio as $\mathrm{IPR} = \sum_n |\psi_n|^4/(\sum_n |\psi_n|^2)^2$. The IPR of a localized state tends to 1 while for extended states it tends to 0. For $\delta > 1$ the system is in a topological phase and we only have states with low IPR inside the energy gap corresponding to the two edges and domain wall states. The domain wall state decays exponential to both sides and on the same sub-lattice due to sub-lattice symmetry. The state on the domain wall hybridizes to the edges when the system is in a topological phase, this causes that part of the state to have weight on the edge, lowering the IPR. Finally, we also plot the amplitude of the domain wall state for $z = 0$ and $z = Z_m$, that is when the domain wall performs one movement on the array (the intensity plot can be seen on Figure 1 of the main text). Dynamics shows that the final state has a phase change of π over the bending curvature.

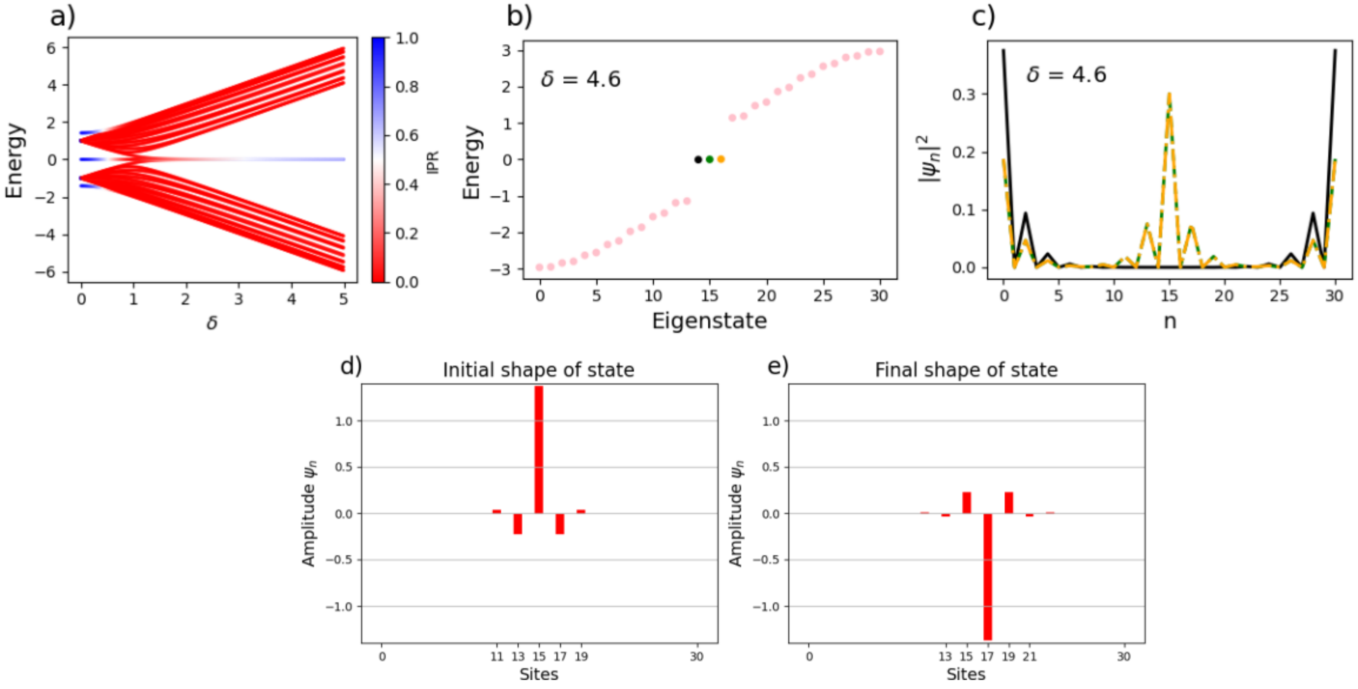

Figure S1. **a)** Energy bands as a function of the coupling relation δ. The inverse participation ratio is represented with a color scale for each state. **b)** Example of the energy spectrum for $\delta = 4.6$. The topological states are highlighted. **c)** Wave-function distribution across the lattice for the highlighted zero energy states shown in b). d) Amplitude profile of the domain wall at $z = 0$. e) Amplitude profile of the domain wall at $z = Z_m$.

We now address the case of a domain wall in a system when only one edge supports a topological state. This can be done by cutting one edge site as shown in Figure S2. From that figure we notice that the domain wall state remains at zero energy but in this case it is hybridized to the only topological edge as depicted in Figure S2 b,c). Interestingly enough, the state can withstand the transfer just like the full topological case.

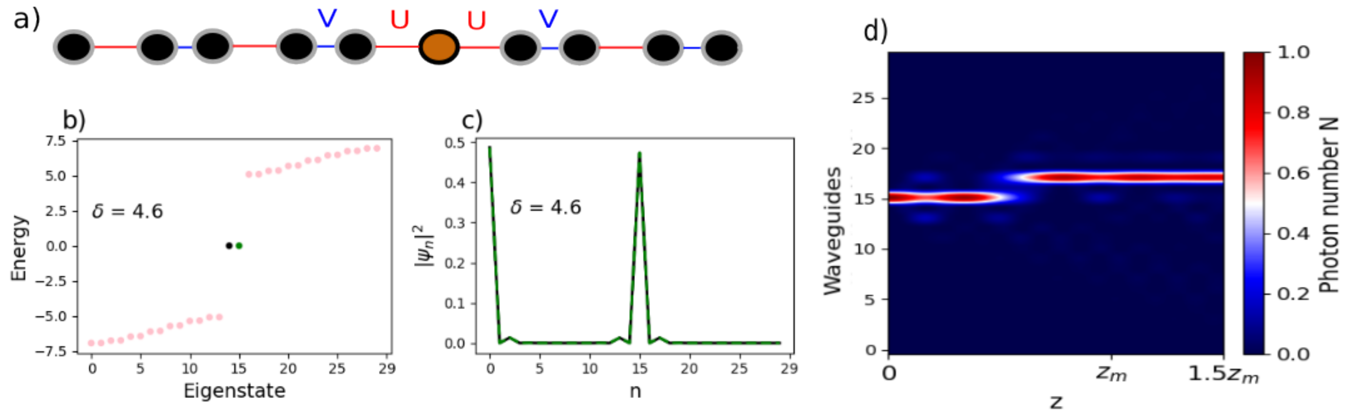

Figure S2. **a)** Shows a scheme of an array with only one topological state on the left edge and none on the other. **b)** Shows the energy spectrum with both the edge (black point) and domain wall (green point) states inside the energy gap. **c)** Shows the squared absolute value of the wave-function of both edge and domain wall states inside the energy gap. **d)** Shows the propagation of light when moving the domain wall.

S2. NUMERICAL METHOD FOR THE EVOLUTION OF QUANTUM STATES OF LIGHT IN TIME-DEPENDENT LINEAR HAMILTONIAN

In order to get the expectation values for the photon number and two-point correlation function (using a time-dependent Hamiltonian), we exploit the linearity of the Hamiltonian and a time-dependent trotter algorithm [1] to compute these quantities. A linear Hamiltonian can be written as $\hat{\mathcal{H}} = \sum_{m,n} H_{mn}\hat{a}_m^\dagger \hat{a}_n$ and, if it is independent of time, the evolution operator is $\hat{U}(t_0, t) = \exp\{-i(t-t_0)\hat{\mathcal{H}}\}$. In the Heisenberg picture and using the BCH formula, the ladder operators fulfill:

$$\hat{a}_j(z) = e^{ib\hat{\mathcal{H}}}\hat{a}_j e^{-ib\hat{\mathcal{H}}} = \sum_n e^{-ibH_{jn}}\hat{a}_n. \tag{1}$$

For time-dependent Hamiltonians we need to use the trotter approximation as:

$$\begin{aligned}\hat{U}(t_0, t_0+\Delta t) &\approx \exp\left\{-i\int_{t_0}^{t_0+\Delta t}\hat{\mathcal{H}}(s)ds\right\} \\ &= \exp\{-B(t_0,\Delta t)_{mn}\,\hat{a}_m^\dagger\hat{a}_n\}\ .\end{aligned} \tag{2}$$

The evolution of the ladder operator in this case in the Heisenberg picture looks as:

$$\begin{aligned}\hat{a}_m(t_0+\Delta t) &= \hat{U}^\dagger(t_0, t_0+\Delta t)\hat{a}_m(t_0)\hat{U}(t_0,t_0+\Delta t) \\ &= \sum_n U_{mn}(t_0,\Delta t)\hat{a}_n(t_0)\ ,\end{aligned} \tag{3}$$

with $U_{mn}(t_0,\Delta t) = \exp\{-i\int_{t_0}^{t_0+\Delta t} H_{mn}(s)ds\}$, a (N,N) matrix with N the size of the lattice. To calculate the evolution of the expectation values for the photon number and two-point correlation function we compute:

$$\begin{aligned}N_{ij}(t_0+\Delta t) &= \langle\psi(t_0+\Delta t)|\,\hat{a}_i^\dagger\hat{a}_j\,|\psi(t_0+\Delta t)\rangle \\ &= \sum_{nm} U_{im}^*(t_0,\Delta t)U_{jn}(t_0,\Delta t)N_{mn}(t_0)\ .\end{aligned} \tag{4}$$

The initial condition $N(t_0)$ is calculated analytically, then the simulation consists of repeatably multiplying the two evolution matrices with the N_{mn} on each time. For $g^{(2)}_{ijkl}$ the recipe is the same but four contractions are needed, one for each index as:

$$g^{(2)}_{ijkl}(t_0+\Delta t) = \sum_{mntp} U_{im}^*U_{jn}U_{kt}^*U_{lp}g^{(2)}_{mntp}(t_0)\ . \tag{5}$$

Again, we just need to calculate the initial condition for the correlation g^2 and the evolution is computed iterating. To calculate the uncertainty we use quadrature $\hat{X}_i^{(1)}$

$$\langle \Delta \hat{X}_i^1 \rangle = \frac{1}{4}\left(\langle \hat{a}_i \hat{a}_i \rangle e^{-2i\phi} + \langle \hat{a}_i^\dagger \hat{a}_i \rangle + \langle \hat{a}_i \hat{a}_i^\dagger \rangle + \langle \hat{a}_i^\dagger \hat{a}_i^\dagger \rangle^{2i\phi} \right) . \tag{6}$$

Here we used $\langle \hat{a}_i \rangle_{sq} = \langle \hat{a}_i^\dagger \rangle_{sq} = 0$, because vacuum squeezed states have zero first momenta. The orthogonal quadrature is obtained analogously. Using the same method as before we can compute the evolution for all these matrices and obtain the evolution for the uncertainty, which corresponds to Eq. (7) of the main text.

S3. OPTIMIZING THE ADIABATIC EVOLUTION

In this section we address the optimal conditions for the modulation and the choice of the final set of parameters in order to have the best transmission of the domain wall. As depicted in Equation (9) of the main text, we have three mayor parameters: A and B which are defined by the coupling between waveguides, Z_m defines the length of the modulation (how long it takes to transfer the state) and the slope of the curvature s. Both the coupling and length of modulation are related to each other, defining the effective propagation of the light. For larger values of couplings, we need shorter modulation lengths as the effective propagation is greater for a given Z_m, meanwhile for lower values of coupling, the opposite case happens. Although there exists many combination of these parameters, not all of them could be used in a realistic experiment. They also do not maximize transmission. For this reason, once the values of A and B are fixed (meaning realistic values of coupling), we focus mainly in the parameters s and Z_m. The transmission curves as a function of s for different values of Z_m are plotted below:

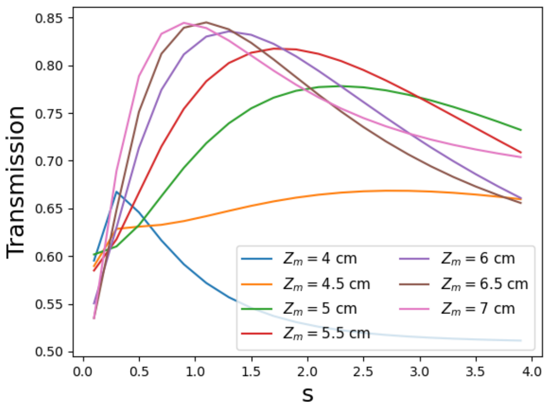

Figure S3. Transmission as a function of the slope s for several modulation lengths Z_m. Lattice parameters: $d_u = 22\mu$m, $d_v = 10\mu$m and $n = 31$.

From Figure S3, we can see that the highest values of transmission are for the highest values of Z_m, reaching values between 80% and 85% of transmission with s between ∼1.0 and 1.5. We chose $s = 1.5$ and $Z_m = 5.5$ cm making a trade-off between modulation length and transmission. Although this set of parameters reaches a slightly lower transmission, it has the advantage that the total length is shorter. Therefore it is more convenient from an experimental point of view, having in mind the actual length of the samples typically used.

[1] D. Poulin, A. Qarry, R. Somma, and F. Verstraete, Quantum Simulation of Time-Dependent Hamiltonians and the Convenient Illusion of Hilbert Space, Physical Review Letters **106**, 170501 (2011), publisher: American Physical Society.
